# Molecular Signatures of a TLR4 Agonist-Adjuvanted HIV-1 Vaccine Candidate in Humans

**DOI:** 10.3389/fimmu.2018.00301

**Published:** 2018-02-26

**Authors:** Jenna Anderson, Thorunn A. Olafsdottir, Sven Kratochvil, Paul F. McKay, Malin Östensson, Josefine Persson, Robin J. Shattock, Ali M. Harandi

**Affiliations:** ^1^Department of Microbiology and Immunology, Institute of Biomedicine, Sahlgrenska Academy, University of Gothenburg, Gothenburg, Sweden; ^2^Department of Medicine, Mucosal Infection and Immunity Group, Section of Virology, Imperial College London, London, United Kingdom

**Keywords:** HIV vaccine, systems vaccinology, transcriptomics, adjuvant, glucopyranosyl lipid adjuvant-aqueous formulation, TLR4

## Abstract

Systems biology approaches have recently provided new insights into the mechanisms of action of human vaccines and adjuvants. Here, we investigated early transcriptional signatures induced in whole blood of healthy subjects following vaccination with a recombinant HIV-1 envelope glycoprotein subunit CN54gp140 adjuvanted with the TLR4 agonist glucopyranosyl lipid adjuvant-aqueous formulation (GLA-AF) and correlated signatures to CN54gp140-specific serum antibody responses. Fourteen healthy volunteers aged 18–45 years were immunized intramuscularly three times at 1-month intervals and whole blood samples were collected at baseline, 6 h, and 1, 3, and 7 days post first immunization. Subtle changes in the transcriptomic profiles were observed following immunization, ranging from over 300 differentially expressed genes (DEGs) at day 1 to nearly 100 DEGs at day 7 following immunization. Functional pathway analysis revealed blood transcription modules (BTMs) related to general cell cycle activation, and innate immune cell activation at early time points, as well as BTMs related to T cells and B cell activation at the later time points post-immunization. Diverse CN54gp140-specific serum antibody responses of the subjects enabled their categorization into high or low responders, at early (<1 month) and late (up to 6 months) time points post vaccination. BTM analyses revealed repression of modules enriched in NK cells, and the mitochondrial electron chain, in individuals with high or sustained antigen-specific antibody responses. However, low responders showed an enhancement of BTMs associated with enrichment in myeloid cells and monocytes as well as integrin cell surface interactions. Flow cytometry analysis of peripheral blood mononuclear cells obtained from the subjects revealed an enhanced frequency of CD56^dim^ NK cells in the majority of vaccines 14 days after vaccination as compared with the baseline. These results emphasize the utility of a systems biology approach to enhance our understanding on the mechanisms of action of TLR4 adjuvanted human vaccines.

## Introduction

Since the onset of the epidemic, more than 70 million people have been infected with human immunodeficiency virus (HIV), and about 35 million people have died of HIV (1). An efficacious vaccine, alone or in combination with other interventions, is widely considered to hold promise for controlling the spread of HIV infection worldwide (2). Despite numerous efforts over the past three decades, an effective anti-HIV vaccine remains elusive. As exemplified in previous vaccine trials [reviewed in Ref. (3)], including the recent encouraging RV144 trial in Thailand (4), inter-individual variation in the immune response to HIV-1 is one of the several challenges for developing a broadly protective anti-HIV vaccine (5).

Antibodies directed against the viral *Env* protein are largely accepted to contribute to protection against HIV infection (6), though the nuanced involvement of different antibody classes or subclasses is unclear. Recent studies have shown that immunization with the stable trimeric recombinant HIV-1 envelope glycoprotein, CN54gp140, induces strong systemic and mucosal immune responses following different immunization routes and strategies (7–9). In particular, immunization with this candidate antigen has induced potent humoral immune responses when adjuvanted with TLR4 agonist adjuvants, such as monophosphoryl lipid A (9) or GLA-AF (glucopyranosol lipid adjuvant-aqueous formulation) (10). These adjuvants are deemed to exert their adjuvanticity, at least in part, by activating the myeloid differentiation factor 88 (MyD88) and toll-interleukin 1 receptor domain-containing adapter inducing interferon-β (TRIF) pathways (11–13). Nevertheless, the magnitude of elicited responses to CN54gp140 adjuvanted with GLA-AF are variable between individuals, the degree to which such variability could be contributed to difference in responsiveness to the adjuvant are unknown.

Early transcriptomics profiling has recently provided new insights into the mode of action of human vaccines and adjuvants (14–16), and has the potential to help elucidate mechanisms underlying diverse individual responses to identical vaccinations. Systems biology was first used to identify gene signatures correlating with immune responses in humans following vaccination with the yellow fever vaccine YF-17D (17). Other studies have since then employed whole blood or peripheral blood mononuclear cell samples to evaluate gene expression signatures of human vaccines and correlate them with vaccine responses [for example (14, 18–20), and as reviewed in Ref. (15)]. Here, we employed whole genome transcriptomics combined with a systems biology approach with the aim of characterizing early molecular signatures induced in the whole blood of healthy volunteers vaccinated with an HIV-1 subunit vaccine candidate, composed of CN54gp140 adjuvanted with the TLR4 agonist GLA-AF. We herein report early transcript signatures and blood transcription modules (BTMs) in the whole blood within 7 days following vaccination. Further, we identified BTMs that were differentially enriched in high vs low serum CN54gp140-specific antibody responders. These results provide new insights into the early blood transcript signatures of TLR4 agonist-adjuvanted HIV-1 envelope glycoprotein vaccine candidates in humans.

## Materials and Methods

### Study Subjects and Vaccine

Healthy male (*n* = 8) or female (*n* = 6) volunteers aged between 18 and 45 and with no history of HIV-1 and HIV-2 infection were enrolled in an open label randomized clinical trial at Imperial College London, UK (study registered at ClinicalTrials.gov under registration no. NCT01966900). Informed written consent was obtained for all volunteers as part of the enrollment process. A recombinant uncleaved clade C HIV-1 envelope gp140 protein (CN54gp140) produced by Polymun Scientific (Klosterneuburg, Austria) to GMP specification, which has previously been reported to be immunogenic in a number of preclinical and clinical studies (7, 8, 21, 22), was used for the clinical trial. All subjects were immunized in the deltoid muscle three times at 1-month intervals with a vaccine candidate composed of 100 µg CN54gp140, adjuvanted with 5 µg GLA-AF. Of the 14 recruited participants, 12 went on to receive additional boosted immunizations at 6 (*n* = 6) or 12 (*n* = 6) months post their third priming immunization. More detailed information concerning study participants and design can be found in Ref. (23). Whole blood samples were taken for RNA analysis at the baseline (day 0) as well as at 6 h and 1, 3, and 7 days following first immunization (reported for this study only). This work was performed in compliance with UK Clinical Trial Regulations and all procedures were approved by the recognized Research Ethics Committee and by the Medicines and Healthcare Products Regulatory Authority.

### RNA Samples

Whole blood samples were collected in PAXgene RNA stabilization tubes (Qiagen, USA) from each participant before vaccination (day 0, 0 h) as well as at 6 h and 1, 3, and 7 days following first vaccination. RNA was extracted using the PAXgene Blood RNA kit, according to the manufacturer’s protocols (Qiagen, USA). Spectrophotometry was used to measure RNA concentration (ND-1000 spectrophotometer, NanoDrop Technologies Inc., USA) and the RNA integrity number (RIN), an indicator of sample quality, was determined using an Agilent 2200 TapeStation and 2100 Expert Software (Agilent Technologies, USA). The RIN for the mRNA extracted from the remaining 66 whole blood samples ranged from 6.2 to 9.0 with a mean of 7.9 ± 0.6 for all extracted samples. Only samples with good quality mRNA, defined as having a 260/280 ratio approximating 2 and RIN >7, were used in microarray analyses.

### Microarray Analysis and Data Acquisition

Whole genome microarray analysis was performed at the Genomics Core Facility, University of Gothenburg, Sweden using an Illumina platform according to the manufacturer’s protocols. Briefly, the Fluorescent Linear Amplification Kit was used for RNA labeling, and quantity and labeling efficiency were verified prior to hybridization of samples to whole genome 8x60k human expression arrays (HumanHT-12 v4 Expression BeadChip kit; Illumina, USA). Post-hybridization scans were performed using an Agilent scanner at 5 µm and image analysis to generate raw data was completed using Feature Extraction software (version 11.5.1.1, Agilent Technologies). The raw data was pre-processed and normalized using the limma package and corrected for background using the “normexp” method in the statistical program R (24). Differentially expressed genes (DEGs) compared to baseline were identified by performing the moderate Student’s *t*-test at each of the time points (*p*-value) followed by further adjustments for multiple testing using the Benjamini–Hochberg method (adjusted *p*-value). Changes in expression at the four time points compared to baseline were calculated by subtracting the normalized expression value of each individual transcript at baseline from that at the specified time point. Following microarray analysis, four samples were excluded from further analyses due to failed cRNA synthesis. In total, 62 high quality mRNA samples were included in further analyses (Figure S1A in Supplementary Material).

### Gene Set Enrichment Analyses

Gene set enrichment analysis (GSEA) using the blood transcription modules identified in Ref. (14) was applied to the data at each individual time point compared to baseline, using the GSEA software from the Broad Institute (25, 26).

Serum samples were obtained during the course of the study and concentrations of CN54gp140-specific serum IgM, IgA, and IgG (including subclasses IgG1, IgG2, IgG3, and IgG4) antibodies were determined as previously described (23). Individuals were classified either as low or high responders based on early CN54gp140-specific IgM (14 days), IgG (28 days), and IgA (28 days) antibody concentrations, as well as on late antigen-specific serum antibody concentrations (168 days for IgA, IgG, IgG1, IgG2, and IgG4; 84 days for IgG3) (Figure S2 in Supplementary Material). These groupings were statistically validated using an unpaired Mann–Whitney test (at least *p* < 0.05 for all). GSEA using BTMs was applied as described above, to individual-level gene expression data for high or low antibody responders compared to baseline, per antibody class or subclass.

### Antibodies

For phenotypic analysis of lymphocyte subsets by multi-color flow cytometry, PBMCs were stained with fluorochrome-conjugated antibodies for the surface markers CD3 (OKT3, Biolegend), CD4 (SK3, Biolegend), CD8 (SK1, Biolegend), CD14 (M5E2, Biolegend), CD19 (HIB19, Biolegend), CD56 (NCAM16.2, BD Biosciences), CD94 (DX22, Biolegend), CD161 (HP-3G10, Biolegend), CRACC (235614, R&D Systems) NKG2A (Z199, Beckman Coulter), NKp80 (MA152, Beckman Coulter), and TCRγδ (IMMU510, Beckman Coulter) as well as for intracellular EOMES (WD1928, eBioscience), granulysin (GNLY) (DH2, Biolegend), NKG7 (2G9, Beckman Coulter), and PLZF (R17-809, BD Biosciences). Intracellular HOPX was detected with an unconjugated primary antibody (rabbit polyclonal, Proteintech) followed by a fluorochrome-labeled F(ab)_2_ anti-rabbit IgG secondary antibody (goat polyclonal, ThermoFisher). Dead cells were identified using a fixable live/dead stain (ThermoFisher).

### Flow Cytometry

Cryopreserved PBMCs from 0 h to 14 days were thawed, washed with cold RPMI-1640 (Gibco) supplemented with 10% FCS (Sigma-Aldrich) and labeled on ice with fluorochrome-conjugated antibodies (Table S1 in Supplementary Material) and live/dead stain in FACS buffer (PBS supplemented with 2% FCS and 2 mM EDTA; all Sigma-Aldrich). Cells were then washed with FACS buffer, fixed at room temperature in PBS containing 2% formaldehyde (Polysciences Inc.), permeabilized at room temperature in PBS with 0.1% Triton X-100 (Sigma-Aldrich) followed by intracellular staining performed in FACS buffer at room temperature. Samples were acquired on an LSRII Fortessa (BD Bioscience) and data analyzed and visualized using FlowJo software (v9.9.4; FlowJo, LLC), and GraphPad PRISM (v5.0). Gating strategy is shown in Figure S3 in Supplementary Material.

## Results

### Overall Changes in Gene Expression in Response to Vaccination

**Figure 1:**
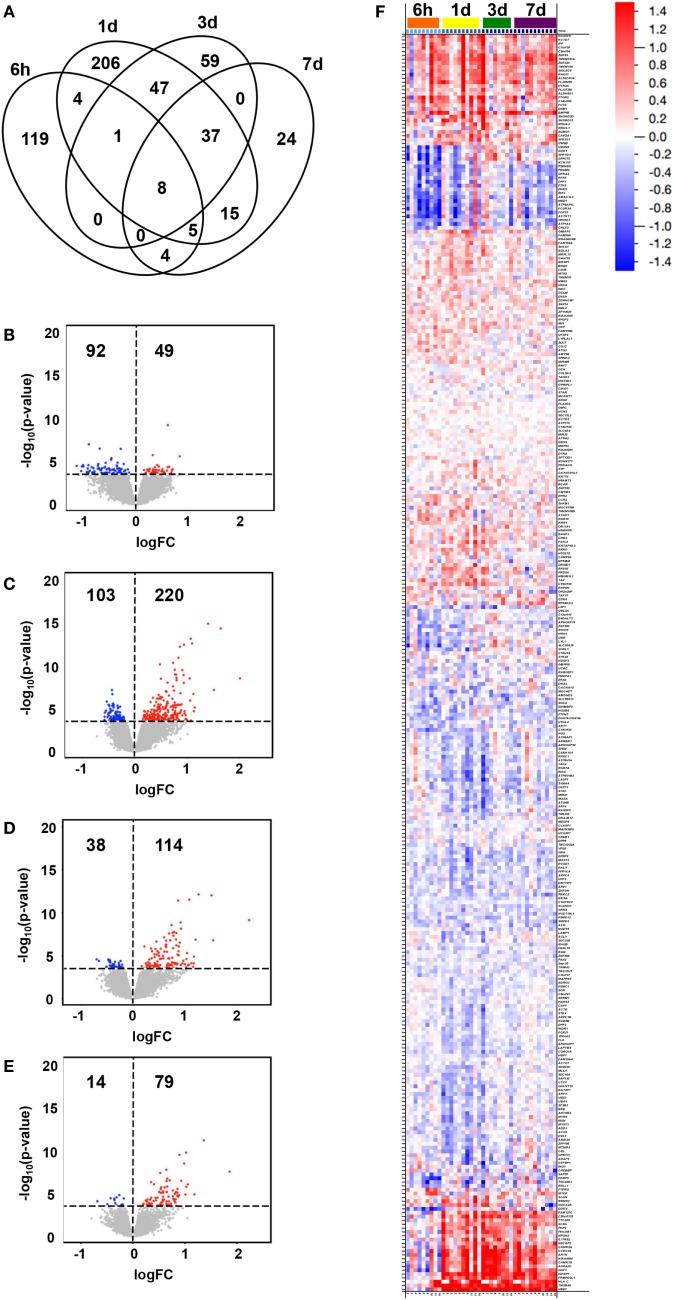
Transcriptional changes in gene expression over time following vaccination. Subjects were intramuscularly immunized with CN54gp140 + GLA. In the Venn diagram, **(A)** the number of unique and shared differentially expressed genes (DEGs) in whole blood at 6 h, 1 day, 3 days, and 7 days after immunization (compared to baseline, 0 h) are shown, while the numbers and magnitudes (*y*-axis) of upregulated (red dots) or downregulated (blue dots) DEGs at 6 h **(B)**, 1 day **(C)**, 3 days **(D)**, and 7 days **(E)**, are depicted in volcano plots. An overview of all DEGs hierarchically clustered and arranged by time, are shown in a heat map **(F)**, according to the color legend (upregulated DEGs in red, downregulated in blue, log_2_FC scale).

Changes in expression at any time point following vaccination compared to baseline (0 h) were identified for 529 genes (adjusted *p*-value < 0.05, Table S2 in Supplementary Material), for which the peak number of uniquely expressed DEGs was observed at 1 day post-first immunization (206 transcripts; Figure 1A). The majority of total DEGs were observed within 24 h post vaccination (329) compared to later time points (59 at 3 days and 24 DEGs at 7 days post-immunization). Of the transcripts uniquely and differentially expressed at the different time points compared to baseline, the lowest number was observed at 7 days post-immunization (24), reflecting a return to baseline within the observational period. The majority of DEGs identified at 6 h were downregulated compared to baseline (92/141, 65%), while at all other time points, the majority of DEGs were predominantly upregulated compared to baseline (220/323, 68% at 1 day; 114/152, 75% at 3 days; and 79/93, 85% at 7 days) (Figures 1B–E).

The magnitude of observed group level transcriptional changes varied between time points compared to baseline (Figures 1B–F), and ranged between −1.132 log_2_FC and 2.220 log_2_FC with the largest spread at 3 days (−0.696–2.220 log_2_FC). A total of 108 DEGs were identified to illustrate the largest fold change compared to baseline (>|0.500| log_2_FC, 67 upregulated and 41 downregulated DEGs) (Figure 1F). Of note, 21 genes appeared to switch from downregulated at 6 h to upregulated compared to baseline at later time points for most individuals. These included genes presumably involved in general response to vaccination, such as those related to tissue regeneration and hemoglobin (e.g., *TMSB4X* and *HBE1*), calcium signaling (e.g., *CAMK2A* and *CAMK1G*), and vesicle-mediated transport (e.g., *NECAP2* and *NRSN2*) as well as specific immune-related genes (e.g., *IL17REL, GDF1*, and *HLA-C*) (27, 28).

### Functional Differences in Transcriptional Profiles following Vaccination

**Figure 2:**
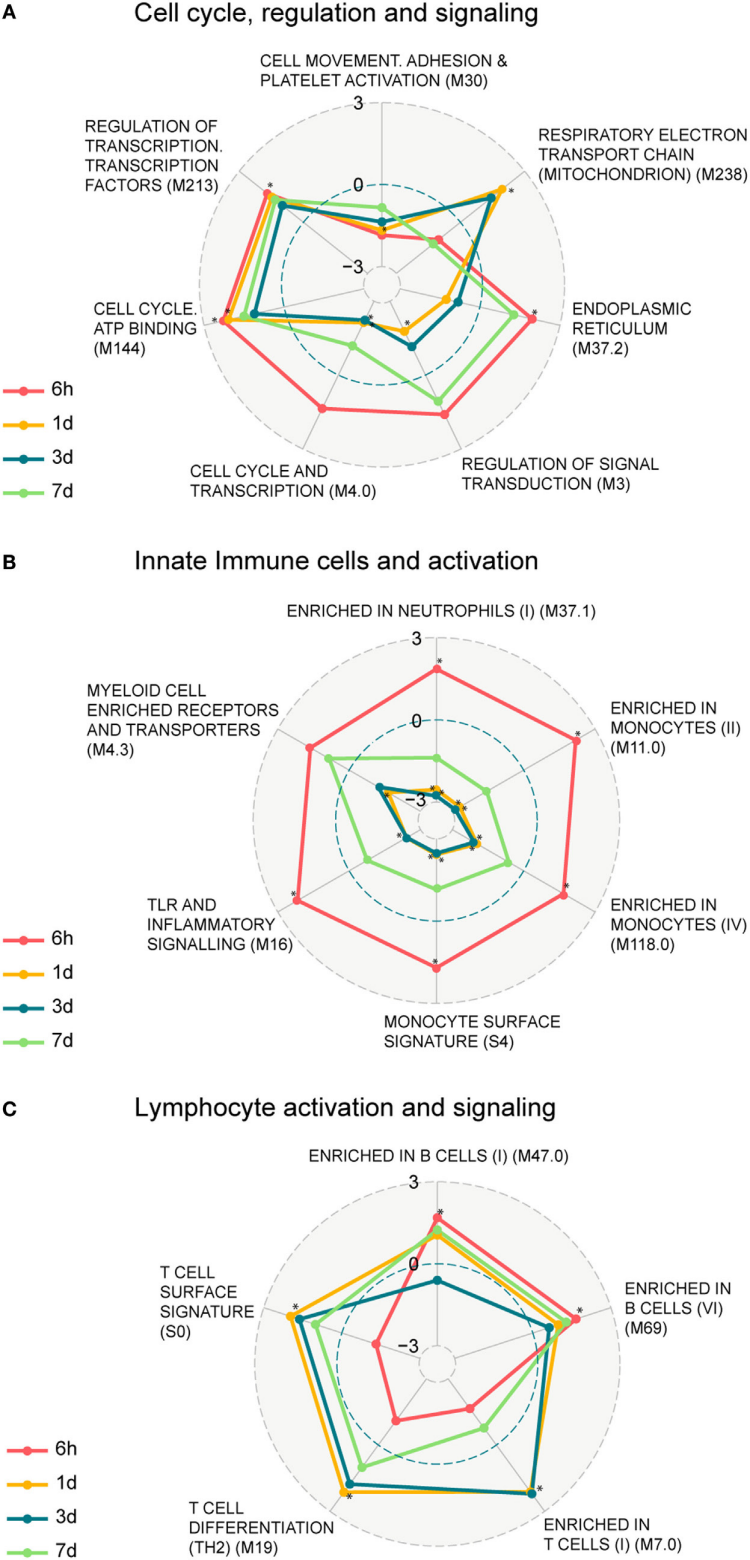
Enrichment of blood transcription modules (BTMs) regulated upon vaccination. Spider web chart indicating enriched BTMs at 6 h (red), 1 day (yellow), 3 days (blue), and 7 days (green). BTMs are functionally divided into: cell cycle regulation and signaling **(A)**, innate immune cells and activation **(B)**, and B and T cell activation and signaling **(C)**. Only BTMs with FDR *q* values < 0.05 at at least one of the four time points (those time points are marked with asterisk) are considered. Points inside the dashed blue circle are negative enrichment and points outside the blue circle are positive enrichment.

We next identified functional pathways perturbed by the vaccination by performing a two-class comparison in GSEA to identify enriched blood transcription modules (BTMs), first presented in Ref. (14). Transcripts were ranked according to a fold change difference relative to the 0 h baseline, and only BTMs with an adjusted *p*-value < 0.05 were considered significant. BTMs with weighted average of logFC for the different BTM forming transcripts being upregulated or downregulated are defined as enhanced or repressed, respectively. The enriched BTMs were divided into the following three main categories: cell cycle regulation and signaling; innate immune cells and activation, and lymphocyte activation and signaling (Figure 2; Table S3 in Supplementary Material). Some of the BTMs involved in cell cycle regulation and signaling were enhanced as early as 6 h following vaccination and remained enhanced until 7 days post vaccination, including regulation of transcription, transcription factors (M213), and cell cycle ATP binding (M144) demonstrating the immediate systemic impact of vaccination on general cell cycle pathways. BTMs representing cell movement, adhesion, and platelet activation (M30) were repressed at all time points (Figure 2A). BTMs related to early immune responses, including toll-like receptors (TLR) and inflammatory signaling (M16), enrichment in neutrophils (I) (M37.1), and several BTMs related to enrichment in monocytes (M11.0, M118.0, S4) were significantly enhanced at 6 h compared to baseline (0 h), whereas most of these BTMs were not found to be significantly enriched at later time points (Figure 2B). BTMs belonging to lymphocyte signaling and activation, including T cell activation and signaling (M7.0, M19, S0), were mainly identified as enhanced at 1 and 3 days post vaccination. By comparison, the B cell modules (M47.0 and M69) were most highly enriched at 6 h followed closely by 1 and 7 days post vaccination (Figure 2C). These results indicate enrichment of BTMs related to cell cycle regulation and signaling as well as those related to innate and adaptive immune responses following a single vaccination with the GLA-adjuvanted CN54gp140 vaccine candidate.

### Early BTM Signatures Induced in Whole Blood Post Vaccination Correlate with Later Serum Antibody Responses

Next, we sought to study whether early transcript signatures correlated with later serum antibody responses. This was achieved by using GSEA to identify enriched BTMs in the transcriptomes of individuals grouped by their CN54gp140-specific antibody responses, at different time point post vaccination relative to the corresponding baseline. Individuals were divided statistically into high and low CN54gp140-specific serum IgM, IgG, or IgA antibody responders based on their serum antibody concentrations and separation into groups was validated using statistical tests as described above (Figure S2 in Supplementary Material).

**Figure 3:**
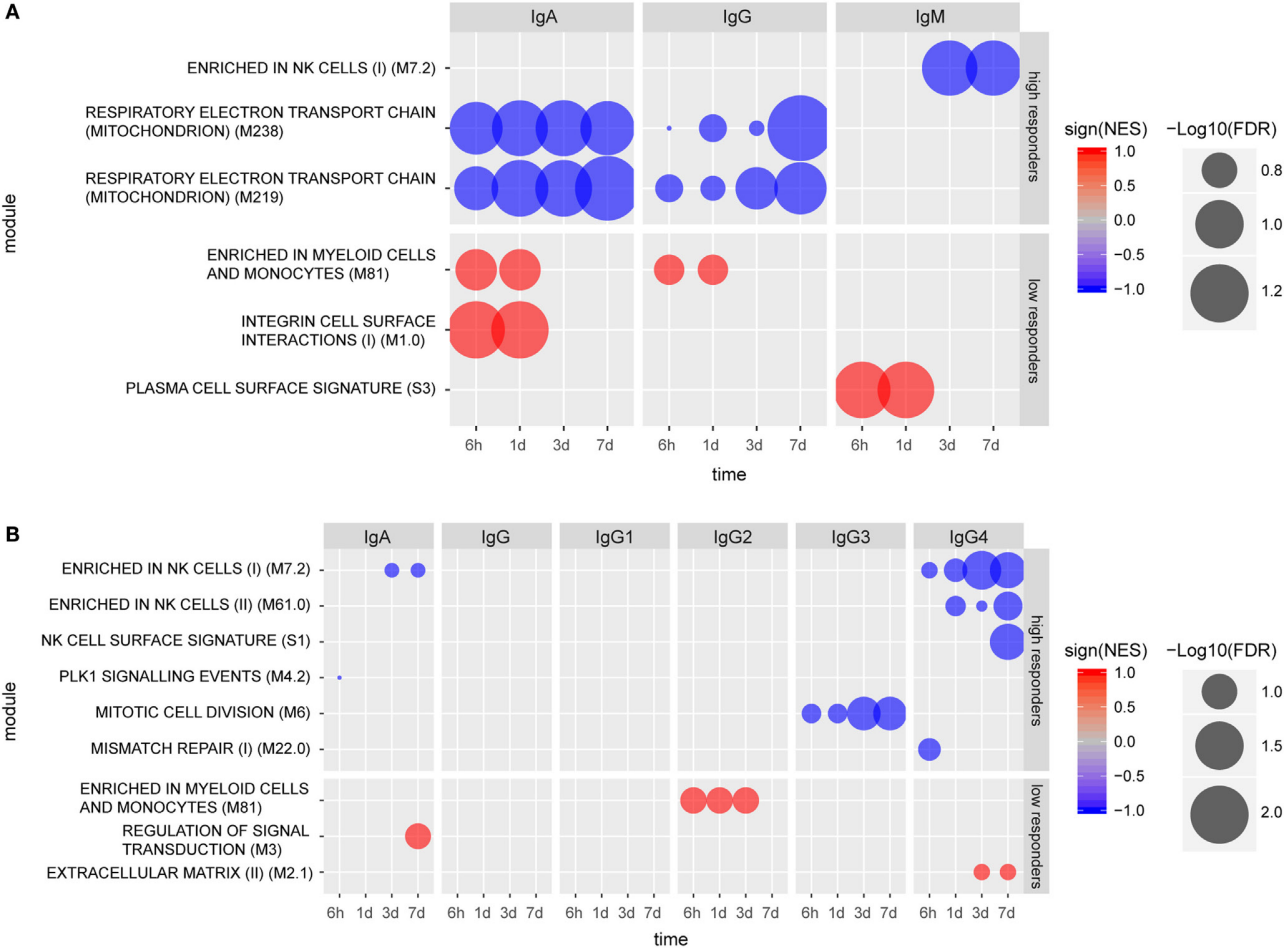
Molecular signatures associated with early **(A)** and late **(B)** humoral responses. Blood transcription modules (BTMs) at early time points (6 h, 1 day, 3 days, and 7 days) associated with IgA (measured at 14 days), IgG (measured at 28 days), and IgM (measured at 28 days) responses in high and low responders **(A)**. BTMs at early time points (6 h, 1 day, 3 days, and 7 days) associating with IgA, IgG, IgG1, IgG2a, IgG3, and IgG4, all measured at 168 days except IgG3 (measured at 84 days) responses in high and low responders **(B)**. Size of the circles indicate level of significance [−log10(FDR)]. Only BTMs with FDR *q* value < 0.25 are included in the plot. Negative association is indicated by blue color and positive association is indicated by red color.

Individuals categorized as high IgM responders (day 14) exhibited a gene signature marked by a repressed enrichment of NK cell-related genes (M7.2) at 3 and 7 days post-vaccination (Figure 3A). Genes including *KLRB1, KLRD1, NKG7, SLAMF7, TBX21*, and *IL2RB* contributed to the core NK cell enrichment and displayed downregulation compared to the baseline. In contrast, gene expression levels for low IgM responders at 6 h and 1 day exhibited enhancement in the BTM for “plasma cell surface signature” (S3) (Figure 3A), including genes *SLC44A1, KCNH1, CAV1, TXNDC15*, and *KRTCAP2*. The repression of two BTMs related to respiratory electron transport chain (mitochondrion) (M219, M238) marked early antigen-specific IgG and IgA responders, while enhancement of BTMs enriched for myeloid cells and monocytes (M81) at 6 h and 1 day post-vaccination were identified only in low IgG or IgA serum responders. Enhancement in the BTM, “integrin cell surface interactions” (M1.0) was also identified as another early BTM signature for low antigen-specific IgA responders.

Vaccinees were also categorized into high and low responders at later time points (up to 6 months post vaccination) based on their individual CN54gp140-specific serum antibody concentrations (Figure S2 in Supplementary Material). Using this analysis, no specific BTMs were identified using the gene expression data from individuals classified as high or low serum IgG or IgG1 responders. However, BTM-based gene signatures were identified which differentiated late high serum IgA, IgG3, and IgG4 responders from low responders (Figure 3B). Notably, three NK cell-related enriched BTMs (M7.2, M61.0, and S1) were significantly repressed in the gene expression profiles from individuals categorized as either late high serum IgA or IgG4 responders, including downregulation of many of the same genes indicated for early high IgM responders. Different BTMs were also significantly enriched post-vaccination for individuals classified as late low IgA, IgG2, and IgG4 responders (Figure 3B). BTM module “Enrichment in myeloid cells and monocytes (M81)”, which was identified as significantly enhanced for low early serum IgG and IgA responders, was also identified as significantly enhanced for late low serum IgG2 antibody responders.

### Enhanced Frequency of CD3^–^CD56^dim^ NK Cells following Vaccination

**Figure 4:**
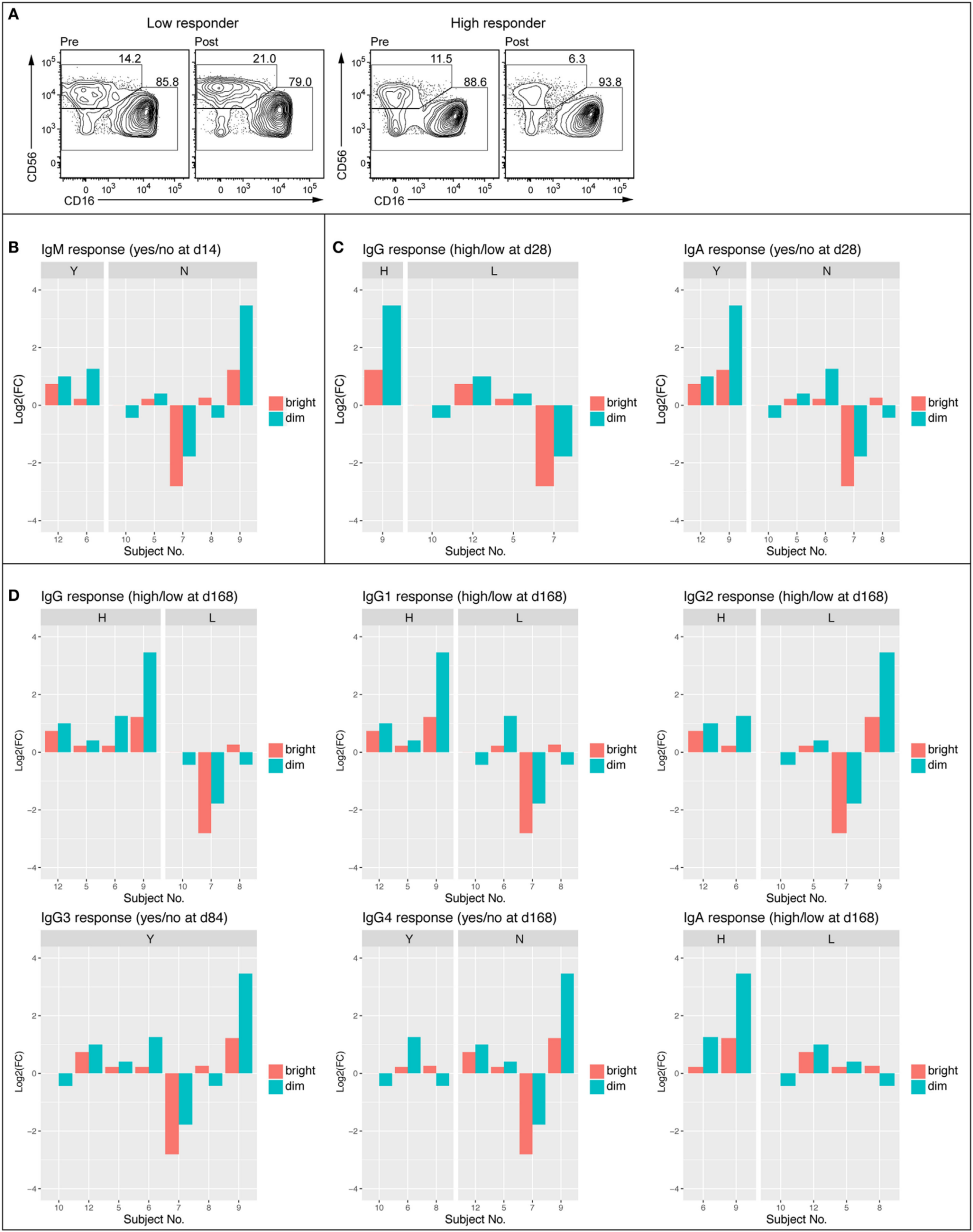
Frequency of CD56^dim^ NK cell was enhanced in high antibody responders 14 days post vaccination. FACS plots showing NK cell populations of representative low (no IgM 14 days and low IgG 168 days) and high (IgM response 14 days and high IgG 168 days) responder before (D0) and 14 days following vaccination **(A)**. Average frequency of the CD56^bright^ and CD56^dim^ NK cell populations at 0 h and 14 days **(B)**. Log2 fold change of CD56^bright^ and CD56^dim^ NK cell population divided into IgM responders (Y) and non-responders (N) Day 14 following vaccination **(C)**. Log2 fold change of CD56^bright^ and CD56^dim^ NK cell populations divided into early (84–168 days) high and low responders (IgG, IgG1, IgG2, IgG3, IgG4, and IgA) **(D)**.

Given tendencies for vaccination-induced enrichment in NK cell-related BTMs and significant associations between such NK cell-related BTMs modules and serum antibody responses, we next examined peripheral blood NK cells by flow cytometry in samples from subjects at 0 h baseline and at 14 days post first vaccination. Samples from intermediate time-points were not available. To determine whether observed changes in NK cell-related BTMs could be attributed to change in NK cell numbers or expression levels of proteins upon vaccination, we stained PBMC for lineage markers, NK cell receptors, and an array of proteins within regulated BTMs. Such proteins included transcription factors EOMES and HOPX, granule constituent’s *GNLY* and NKG7, as well as surface receptors CD161 (*KLRB1*), NKG2A (*KLRC1*), NKG2D (*KLRD1*), NKp80 (*KLRF1*), and CRACC (*SLAMF7*). Results showed an overall trend of increase in the frequency of CD3^−^CD56^dim^ NK cell population at 14 days post vaccination compared to the 0 h baseline (Figure 4). Levels of EOMES, HOPX, GNLY, NKG7, CD161, NKG2A, NKG2D, NKp80, and CRACC were not increased in CD3^–^CD56^dim^ NK cells at 14 days post vaccination (data not shown). In the limited number of analyzed samples, frequency of CD3^–^CD56^dim^ NK cell population in the blood of high antibody responder subjects was increased on 14 days post vaccination compared to the 0 h baseline (Figure 4A). This was observed for all of limited number of the early IgM (Figure 4B), IgG, and IgA (Figure 4C) high responders, whereas three out of four (IgM), two out of four (IgG), and three out of five (IgA) of the low responders demonstrated a decrease of the CD3^–^CD56^dim^ NK cell phenotype. Similar trends were observed when changes in NK cells frequencies between baseline and 14 days post vaccination were studied in vaccines defined as high versus low responders at later time points (Figure 4D).

## Discussion

Herein, we employed a whole genome transcriptomics analysis combined with a systems biology approach to pinpoint early transcriptional signatures in whole blood of healthy volunteers vaccinated with the TLR4 agonist GLA-AF adjuvanted CN54gp140 HIV-1 vaccine candidate. We identified DEGs and BTM signatures of the whole blood response to the vaccine candidate up to 7 days following vaccination, and the BTMs that showed correlation with early and late CN54gp140-specific antibody responses.

We observed an increase in the number of DEGs compared to the baseline as early as 6 h post-vaccination, which peaked at 1 and 3 days followed by a decline to the expression levels comparable to that of baseline by 7 days. The observed kinetics of the transcriptional changes in whole blood is in line with previous studies (15, 29). Notably, transcriptional analysis of the TLR4 agonist GLA adjuvant in mice revealed minimal gene regulation in blood compared with the site of injection (muscle) and draining lymph nodes (13), highlighting one of the major limitations of our and most human studies. A group of 21 genes were identified that demonstrated the greatest fold changes compared to baseline, and additionally appeared to switch from downregulated compared to baseline at 6 h to upregulated compared to baseline at later time points, for most vaccinated individuals. Several of these identified genes (such as *TMSB4X* and *HBE1*) were likely involved in the general response to vaccination, including hemoglobin genes and genes related to tissue regeneration. Of interest, all except three of the individuals had significantly increased HLA-C expression at D1, D3, and D7 relative to the baseline. HLA-C belongs to the classical MHC molecules with least variability and recently it has been shown to interact with the Env protein of HIV (30), the antigen component of the vaccine used in our study. HLA-C is known to interact with two main ligands, the CD8 T cell receptor and even more efficiently with NK cell receptors in an allele-specific manner, including: NKG2A/C immunoglobulin receptors (KIRs) and, to a lower extent, leukocyte immunoglobulin receptors (31, 32).

The functional pathway analysis of transcriptomics data with GSEA revealed BTMs related to general cell cycle activation, and innate immune cell activation at early time points, as well as BTMSs related to T cells, and B cell activation at the later time points. BTM offers an unprecedented possibility to functionally assess transcriptomic changes related to immunological responses in human blood (14). By statistically categorizing individual vaccines into high or low responder groups based on their CN54gp140-specific serum antibody levels, we identified several BTMs that were significantly enriched in either high or low responders for serum antibody classes and subclasses. In particular, we identified a repression in BTM modules related to NK cells, especially at 3 and 7 days post-vaccination, for high serum IgM, IgA, and IgG4 antibody responders. Our finding is somewhat similar to a recent report, where the BTMs enriched in NK cells and NK cell surface signatures (M7.2, M61.0, and M61.2) were negatively associated with antibody titers in subjects that received the malaria vaccine candidate RTS,S (33) at day 56 of their prime-boost regiment. Collectively, these data may suggest an involvement of NK cells in negatively regulating humoral responses (34).

Notwithstanding, the flow cytometry analyses of the subjects’ PBMCs showed an increase in the frequency of CD3^–^CD56^dim^ NK cells for 14 days post vaccination relative to the 0 h baseline. Unfortunately, the paucity of cellular samples from the subjects for 1–7 days post vaccination prevented our further study of this phenomenon. It is tempting to speculate that the repression of BTMs related to NK cells observed in the first 7 days post-vaccination reflects NK cells leaving the circulation early in the response. Given that NK cells are short lived, the enhanced frequency of NK cells for 14 days post vaccination is presumably attributed to secondary induction of NK cell differentiation processes in response to vaccination. NK cells have been shown to inhibit generation of long-lived memory B cells by suppressing follicular helper T cells during the first days of infection in mice (34). NK cells have previously been suggested to play an important role in the adjuvanticity of GLA-AF and GLA in squalene emulsion (SE) *via* rapid production of IFN-γ (35). It has also been hypothesized that vaccine-induced HIV-specific antibody-dependent cellular cytotoxicity mediated by healthy NK cell activity could help in preventing HIV infection (29). The vast majority of NK cells in the circulation is CD56^dim^ NK cells and considered cytotoxic, while around 10% are CD56^bright^ NK cells and known to produce cytokines (36, 37). Vaccination with the inactivated influenza vaccine has previously been shown to downregulate membrane surface NKp46 cells on CD3^−^CD56^dim^ NK cells and increase NK cell-derived IFN-γ expression (38). Additionally, CD56^bright^ NK cells have been shown to increase following vaccination with a hepatitis B DNA vaccine, with significantly increased expression of CD244 and NKG2D, and correlating with specific T cell responses (39). However, NK cells are associated with both positive and negative regulation of humoral responses (40) and hence it is unclear if the observed repression in NK cells related BTMs has a direct causal role in enhancing humoral response to CN54gp140 adjuvanted with GLA, or is a surrogate of other regulatory events.

In this respect, it is intriguing that low early serum IgG and IgA responders were associated with the BTMs “Enrichment in myeloid cells and monocytes (M81).” The observed enhancement of the BTMs for myeloid cells over those of NK cells in low responders may suggest potential differences in antigen handling and processing between low and high responders. Further, the enhancement in BTMs related to plasma cell enrichment in the low responders warrants further studies to pinpoint whether short-lived plasmablasts are being generated in preference to long-lived plasma cells in the low antibody responders.

In line with the enriched upregulation of early plasma cell signature at 6 h and 1 day observed in the IgM antibody low responders in our study, the yellow fever vaccine showed an inverse correlation of antibody responses with plasma cell and B cell BTMs. Conversely, the immune responses to the trivalent influenza vaccine (TIV) and the quadrivalent meningococcal conjugate vaccine showed a positive correlation to those BTMs. Recently, significant correlations between the vaccine-induced antibody responses and plasmablast signatures following influenza vaccination (20) and interferon signaling following yellow fever and live attenuated influenza vaccinations (17, 20) were reported. These transcript signatures, however, did not show a significant correlation with the vaccine-induced antibody responses in our dataset. This is presumably explained, at least in part, by different mechanisms of action of the live attenuated vaccines and the TLR4 agonist-adjuvanted protein vaccines. In keeping with this notion, Li et al. reported different transcript signatures associating with vaccine-induced immune responses when five human vaccines of different modalities were compared (14). Therefore, it is plausible that while some of the blood transcript signatures are shared among different human vaccines, vaccines with different mechanisms of action may possess differential transcript signatures. Notwithstanding, we could not nevertheless rule out the possibility that some of the identified BTMs, e.g., the IgA correlating BTMs “expression of respiratory electron transport chain” and “elevated integrin cell surface interaction” merely play a surrogate rather than a causal role in the antibody responses. Further studies are needed to pinpoint the possible role of correlating BTMs in the development and/or durability of antibody responses induced following vaccination in humans.

Altogether, we herein report potential transcript and BTM signatures of human whole blood in response to vaccination with the TLR4 agonist-adjuvanted CN54gp140 anti-HIV vaccine candidate. However, a few caveats should be applied to our findings. First, the small sample size suggests interesting observations that would require the execution of larger clinical studies to provide a greater statistical power. Second, PBMC samples from days 1–7 were not available for detailed analysis of changes in the number and phenotype of circulating cells, thus observed transcriptomic changes may reflect differences in mRNA in circulating cells and/or influx/efflux of cellular populations in the systemic circulation. Nevertheless, these results provide new information on the candidate transcript signatures that may have potential as early biomarkers of antibody responses induced by TLR4 agonist-adjuvanted HIV-1 Env vaccine candidates, and support the potential of systems approaches in providing new insights into underlying mechanisms of individual variation in response to vaccines in humans.

## Ethics Statement

This work was performed in compliance with UK Clinical Trial Regulations and all procedures were approved by the recognized Research Ethics Committee and by the Medicines and Healthcare Products Regulatory Authority. The clinical trial study is registered at ClinicalTrials.gov under registration no. NCT01966900.

## Author Contributions

AH and RS conceived the project and designed the experiments. JA, TO, and JP conducted the whole blood RNA sample preparation and quality control, and together with MÖ and AH analyzed the transcriptomics data, and composed the manuscript. SK and PM executed the serology experiments and interpreted the data. All the authors provided critical feedback on the manuscript prior to publication and have agreed to the final content.

## Conflict of Interest Statement

The authors declare that the research was conducted in the absence of any commercial or financial relationships that could be construed as a potential conflict of interest.
