# Supplementary material for: Molecular Signatures of a TLR4 Agonist-Adjuvanted HIV-1 Vaccine Candidate in Humans
Source: Front Immunol. 2018 Feb 26;9:301. doi: 10.3389/fimmu.2018.00301 (PMC5834766; doi:10.3389/fimmu.2018.00301)
Supplement: Supplementary file 1 [file Table_1.PDF]

Supplementary table 1: Antibodies used for FACS staining of cryopreserved PBMCs.

Epitope	Fluorochrome	Clone	Dilution	Source
CD3	BV785	OKT3	1:100	Biolegend
CD4	BV510	SK3	1:100	Biolegend
CD8	BV711	SK1	1:400	Biolegend
CD14	BV510	M5E2	1:100	Biolegend
CD16	AlexaFluor700	3G8	1:200	BD Bioscience
CD19	BV510	HIB19	1:100	Biolegend
CD56	BUV395	NCAM16.2	1:50	BD Bioscience
CD94	APC	DX22	1:25	Biolegend
NKG2A	PE-Cy7	Z199	1:50	Beckman Coulter
NKp80	Biotin	REA845	1:50	Miltenyi
TCRγδ	PE-Cy5.5	IMMU510	1:50	Beckman Coulter
Granulysin	AlexaFluor647	DH2	1:50	Biolegend
NKG7	PE	2G9	1:100	Beckman Coulter
PLZF	PE-CF594	R17-809	1:1000	BD Bioscience
Streptavidin	Qdot605	n.a.	1:200	Thermo Fisher
Live/Dead	Aqua	n.a.	1:1000	Thermo Fisher
