# Supplementary figures and images for: Molecular Signatures of a TLR4 Agonist-Adjuvanted HIV-1 Vaccine Candidate in Humans
Source: Front Immunol. 2018 Feb 26;9:301. doi: 10.3389/fimmu.2018.00301 (PMC5834766; doi:10.3389/fimmu.2018.00301)

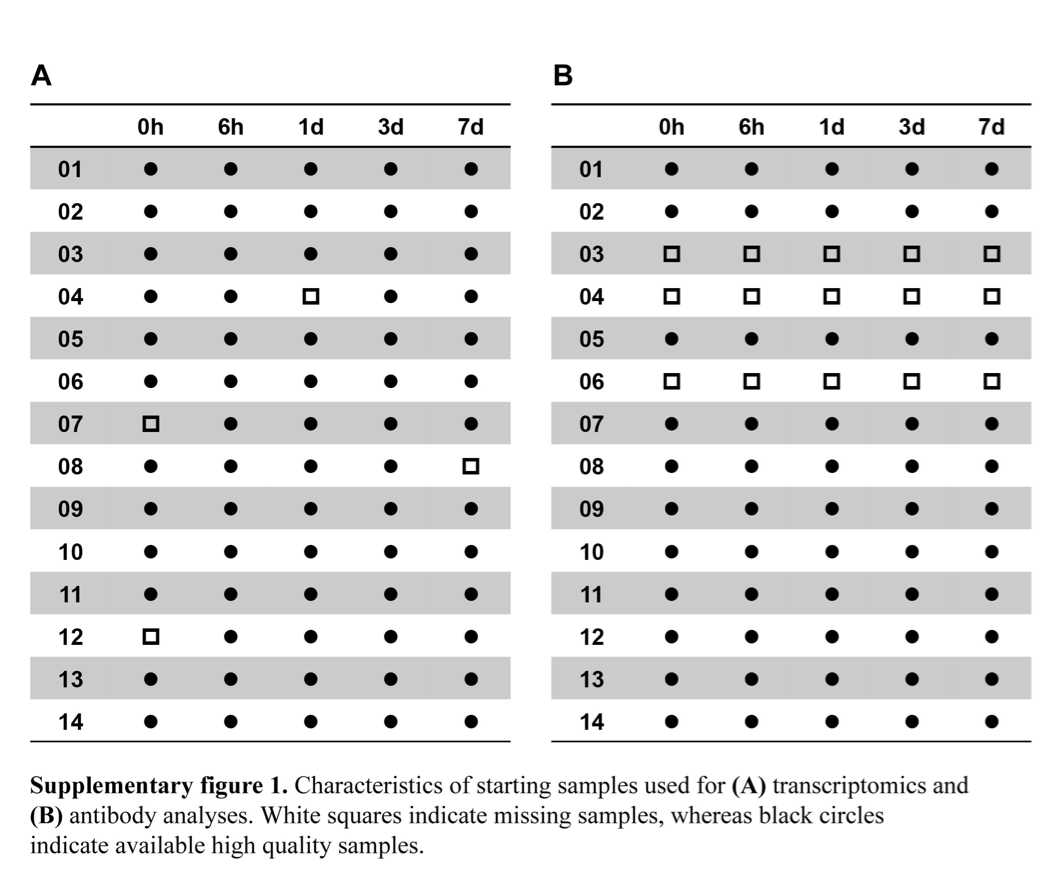

Supplement: Supplementary file 4 [file Image_1.TIFF]

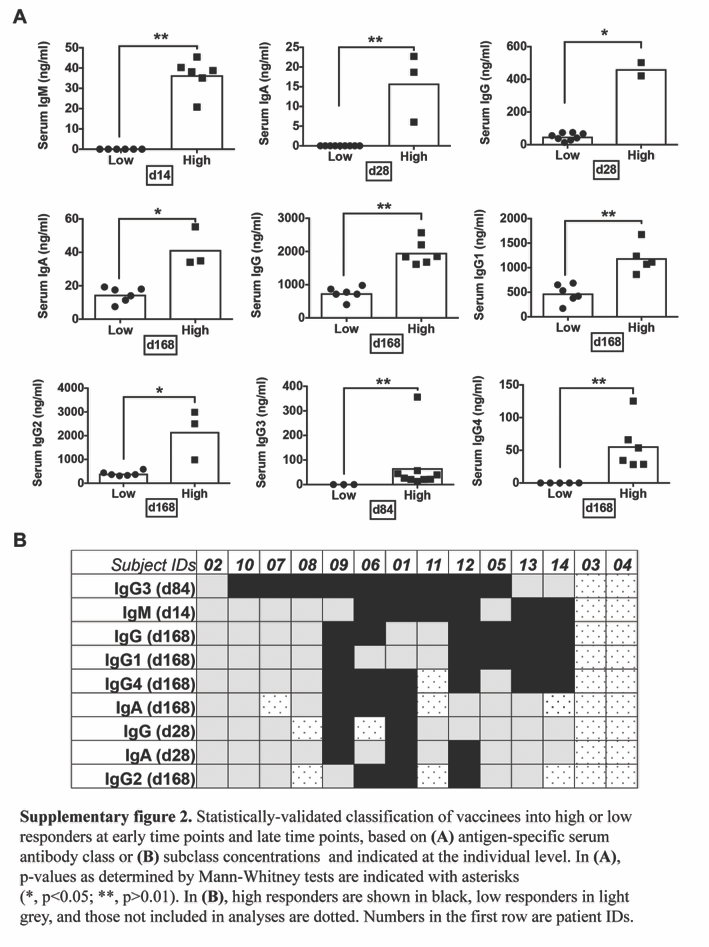

Supplement: Supplementary file 5 [file Image_2.TIFF]

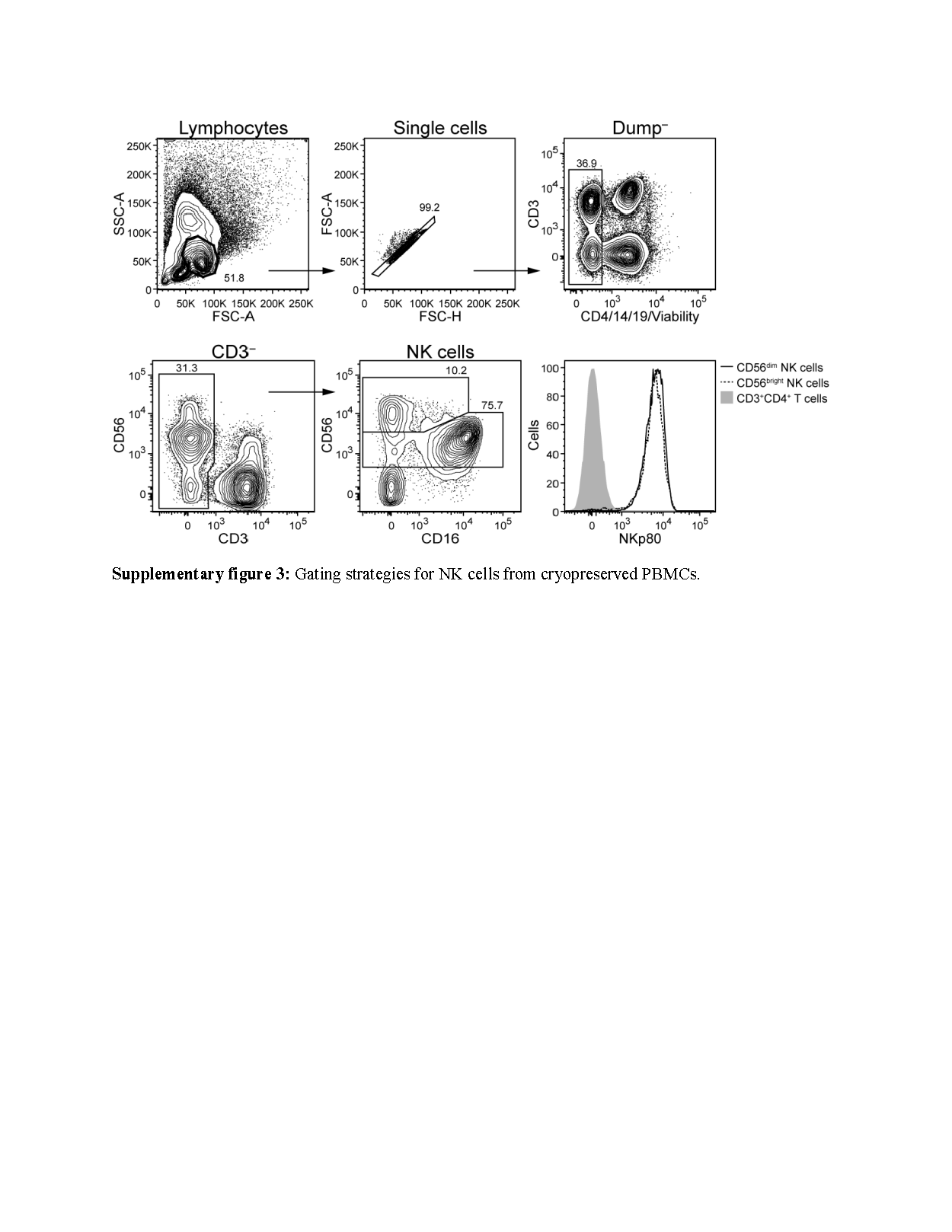

Supplement: Supplementary file 6 [file Image_3.TIFF]
